# Knowledge and Awareness Among Parents of Pediatric Patients Regarding the Complications of Recurrent Adenotonsillitis and Its Surgical Treatment

**DOI:** 10.7759/cureus.20402

**Published:** 2021-12-14

**Authors:** Walaa A Felemban, Rayan Alhussaini, Abdullah F Essa, Alaa A Felemban, Jebreel M Fallatah

**Affiliations:** 1 Otolaryngology-Head and Neck Surgery, Ohud Hospital, Medina, SAU; 2 Otolaryngology-Head and Neck Surgery, Prince Mohammed Bin Abdulaziz Hospital, National Guard Health Affairs, Medina, SAU; 3 Family Medicine, Prince Mohammed Bin Abdulaziz Hospital, National Guard Health Affairs, Medina, SAU; 4 Family Medicine, Eastern al-Jarf Primary Healthcare Center, Medina, SAU; 5 Family Medicine, Bab El Majedi Medical Center, Medina, SAU

**Keywords:** complications, knowledge, parents, children, adenotonsillectomy

## Abstract

Background

Given that parents act as both decision-makers and caregivers for their children, focusing on their experiences in managing their child's complex postoperative recovery is important.

Objective

To evaluate the parents’ awareness regarding adenotonsillar diseases and post-adenotonsillectomy complications in their children.

Methods

This was a cross-sectional descriptive study involving parents and guardians of pediatric patients aged 1-14 years scheduled to undergo adenotonsillectomy according to Paradise Criteria at Ohud Hospital, Al Madinah. A well-constructed, self-administered questionnaire, including questions regarding sociodemographic characteristics and those assessing the knowledge of parents/guardians regarding adenotonsillar diseases and post-adenotonsillectomy complications, was distributed to all participants.

Results

This study included 294 parents (mean ± standard deviation of age, 33.0 ± 6.9 years; range, 18 and 55 years), more than half of whom were males (153, 52%). Overall, 127 parents (43.2%) had a poor level of knowledge regarding the complications of recurrent adenotonsillitis and its surgical treatment. University/postgraduate parents were more knowledgeable than lower educated parents (p < 0.001). Participants with a family history of recurrent adenotonsillectomy were more knowledgeable than those who had no such history (p < 0.001 ).

Conclusion

The parents/guardians of children scheduled to undergo adenotonsillectomy had insufficient knowledge regarding the complications of recurrent adenotonsillitis and its surgical treatment, necessitating health education among such a demographic.

## Introduction

The adenoids and tonsils are part of the lymphatic system that advantageously clears out sources of infection coming to the body through the mouth and nose and maintains the homeostasis of body fluids [1]. Additionally, the tonsils and adenoids possess fundamental functions in humoral and cellular immune systems and are considered important organs of the mucosal immune defense system against antigens invading through the aerodigestive tract [2].

Adenotonsillar disease impacts the adenoids, tonsils, or both; can present as hypertrophy, infection, or both; and are rarely neoplastic [3]. Tonsillitis is a common condition among pediatric and otolaryngology cases. Its management often involves administrating empirical antibiotics without waiting for culture reports. However, the increasing rate of resistance to many organisms resulting from antibiotic misuse due to β-lactamase production and other factors has promoted failure in medical therapy in some occasions, which causes recurrent or chronic tonsillitis [4].

The enlargement of the adenoids and tonsils causes obstruction to breathing and leads to snoring and disturbed sleep in the form of frequent awakening, restless sleep, nightmares, bedwetting, mood changes, excessive sleepiness, and in some cases sleep apnea [5-7]. Some orthodontists suggest that enlarged tonsils and adenoids cause chronic mouth breathing which, in turn, causes improper teeth alignment and malocclusion [8]. Chronic infection and enlargement of the adenoids may promote sinusitis or nasal obstruction and/or may impact the eustachian tube, causing chronic ear infections [9].

Recurrent tonsillitis is defined as more than seven attacks within one year, more than five attacks a year within a two-year period, or more than three attacks a year within a three-year period [10].

Adenotonsillectomy, one of the most common surgical procedures in children, is commonly performed as a day-case surgery. Despite the declining number of tonsillectomies performed over the last years, they still account for 20% of all surgeries performed by otolaryngologists [11].

The most common indications for adenotonsillectomy include recurrent infections, peritonsillar abscess, hypertrophy of the tonsils, and adenoid and obstructive sleep apnea [12-13]. Other indications for adenotonsillectomy include a tumor in the throat or nasal passage, recurrent ear infections, hearing loss, chronic sinusitis, or bad mouth breathing [14-15].

Adenotonsillectomy has been associated with morbidity, including possible hospitalization, risks of anesthesia, prolonged throat pain, infection, postoperative nausea and vomiting, delayed feeding, voice changes, bleeding, respiratory decompensation, subglottic stenosis, and rarely death [16-17].

Determinants for the increased risk of postoperative complications include young age (< 3 years), obesity, airway anomalies, genetic diseases such as Down syndrome, neuromuscular disease, and craniofacial abnormalities [18].

Given that parents act as both decision-makers and caregivers in pediatric cases, it is therefore important to focus on their experiences in managing their child's complex postoperative recovery in order for healthcare providers to better understand and attend to unique patient/parent needs. Various studies have evaluated parents’ awareness regarding adenotonsillectomy in children suffering from upper airway obstruction. This study was designed to evaluate the parents’ awareness regarding adenotonsillar diseases and post-adenotonsillectomy complications in their children.

## Materials and methods

A cross-sectional descriptive study was conducted in Al Madinah, situated in the Hejaz region of Western Saudi Arabia. It has an area of 151,990 km² and has a population of 2,132,679 according to the 2017 census [19].

The target population of this study were parents and guardians of pediatric patients aged 1-14 years scheduled to undergo adenotonsillectomy indicated for recurrent chronic adenotonsillitis or pediatric obstructive sleep apnea at Ohud hospital, which is the only governmental hospital at Al Madinah that has an otolaryngology department. Based on data from the patient affairs department, medical coordination office, operations room manager, and the otolaryngology department, a total of 1014 pediatric patients underwent adenotonsillectomy from 1 January 2018 to 31 December 2018 at Ohud Hospital. 

The inclusion criteria were parents and guardians (regardless of age, nationality, and sex) of pediatric patients aged 1-14 years scheduled to undergo adenotonsillectomy indicated for recurrent chronic adenotonsillitis or pediatric obstructive sleep apnea. Parents/guardians of patients aged below one year and above 14 years with cleft palate, craniofacial anomalies, bleeding or coagulation defects, and congenital anomalies and syndromic features were excluded.

The sample size was calculated using the Epi Info™ statistical software for epidemiology developed by the CDC. The following assumptions were utilized: population size: 1014, expected frequency: 50%, acceptable margin of error: 5%, and confidence interval (CI): 95%. Accordingly, the minimum sample size was 279. A total of 300 subjects were invited to participate in this study to avoid none or incomplete responses.

All patients undergoing adenotonsillectomy according to Paradise Criteria were consecutively admitted into the study one day before surgery. All cases were admitted to the same hospital and treated by different otorhinolaryngologists. Patients were already examined in the outpatient department and scheduled for surgery.

A well-constructed, self-administered questionnaire was distributed to all participants. The questionnaire consisted of two parts. The first part included questions regarding sociodemographic characteristics such as participant's age, sex, nationality, educational level, and family history of recurrent adenotonsillitis, while the second part measured the knowledge of parents whose children were scheduled to undergo adenotonsillectomy and their awareness regarding the role of the adenoid and tonsils in the body and their protective mechanisms, the nature of the disease, complications if neglected and left untreated, and the risks and complications of the surgery.

The second part of the questionnaire had been written in English and comprised 20 questions with three possible answers, namely "Yes", "No", or "I do not know". The questionnaire was translated into Arabic by a certified translation office and then translated back into English. Two otolaryngologists revised the translated questionnaire to suit the local Arabic terminology used by physicians, patients, and participants.

Questionnaires were distributed by the researcher and nurses at the admission office upon patient admission the day before the surgery during morning working hours and collected at the same sitting by the researcher to increase the response rate and save time.

Ethics approval for this study was obtained from the Regional Research and Ethics Committee at Ohud Hospital with approval number ERC-08072019-280. The consent form, which explained the purpose of this study, was included in the self-administered questionnaire. Anonymity was assured, and confidentiality of the data was confirmed. This study was self-funded.

Data entry and statistical analysis

All data were verified by hand and then coded and entered into a personal computer using the double-entry method to decrease date entry errors. Statistical analysis was performed using IBM SPSS Statistics for Windows, Version 25.0 (Released 2017, IBM Corp., Armonk, New York). Categorical variables were described as frequencies and percentages, whereas numerical variables were described as mean and standard deviation. Regarding knowledge statements, correct answers were assigned a score of “1”, whereas wrong answers were assigned a score of “0”. The total score and its percentage were computed. Participants who scored below 50%, between 50% and <75%, and >75% were considered as having “poor knowledge,” “fair knowledge,” and “good knowledge”, respectively. The Chi-square test was used to determine the association between two categorical variables whereas one-way analysis of variance was utilized to determine differences in the mean of a continuous variable between more than two groups. Statistical significance was set at p ˂ 0.05.

## Results

This study included 294 parents (mean ± standard deviation of age, 33.0 ± 6.9 years; range, 18 and 55 years), more than half of whom were males (153, 52%), and a majority of whom were Saudi nationals (276; 93.9%). Regarding their educational level, the majority of the participants were university graduates or above (251; 85.4%) (Table 1).

**Table 1:** Personal characteristics of the participants

Personal characteristics of the participants	
Age	
Range	18-55
Mean ± SD	33.0 ± 6.9
Sex	
Male (No; %)	153; 52.0
Female (No; %)	141; 48.0
Nationality	
Saudi (No; %)	276; 93.9
Non-Saudi (No; %)	18; 6.1
Educational level	
Secondary school/below (No; %)	43; 14.6
University/above (No; %)	251; 85.4

**Figure 1:**
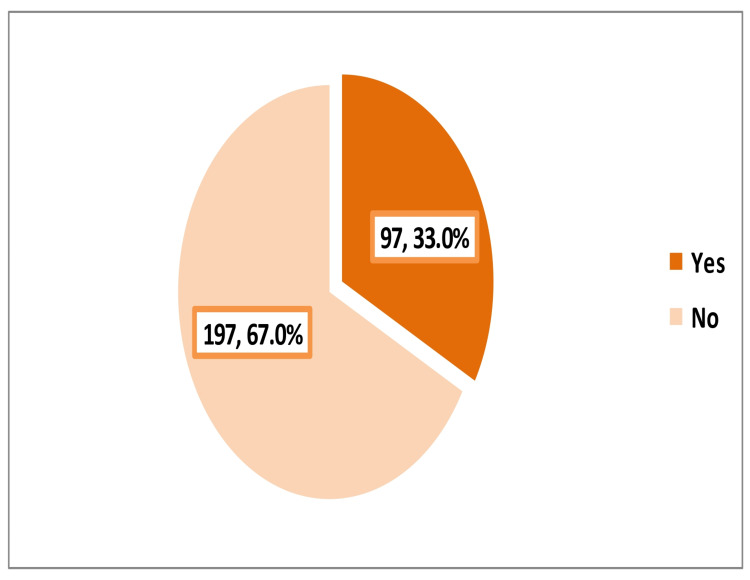
Family history of recurrent adenotonsillectomy among the participated parents

Family history of recurrent adenotonsillectomy among participating parents was observed in almost a third of the participants (97; 33%) (Figure 1).

Table 2 summarizes the responses of participants to knowledge statements regarding the complications of recurrent adenotonsillitis and its surgical treatment. Notably, the majority of the participants (250; 85%) were able to recognize that snoring was associated with tonsils and adenoid hypertrophy and enlarged tonsils and that adenoid hypertrophy can affect the tone of the voice (nasal tone). Most of them (228; 77.6%) understood that recurrent tonsillitis and adenoid infection can cause enlarged tonsils and adenoid hypertrophy and that adenotonsillitis can cause bad odor and smell of the mouth (halitosis) (218; 74.1%). Almost two-thirds of the participants understood that the adenoid and tonsillar hypertrophy causes pediatric sleep apnea (194; 66%) and that adenotonsillectomy can improve otitis media (188; 63.9%). However, around one-fourth of the participants were able to recognize that gastroesophageal reflux disease can cause recurrent adenotonsillitis (79; 26.9%), chronic neglected adenotonsillitis can cause pulmonary hypertension, cor pulmonale, and heart failure (76; 25.9%), and adenotonsillitis can cause dental caries (75; 25.5%).

**Table 2:** Responses of participants to the knowledge statements regarding the complications of recurrent adenotonsillitis and its surgical treatment

	Right answer		
	Response	No	%
Adenoid and tonsils are considered as lymphatic tissue	Yes	160	54.4
Recurrent tonsillitis and adenoid infection can cause enlarged tonsils and adenoid hypertrophy	Yes	228	77.6
Adenoid hypertrophy may affect growth and development	Yes	121	41.2
Facial growth can be affected by adenoid hypertrophy (e.g., Jaw protrusion , teeth malocclusion )	Yes	150	51.0
Adenotonsillectomy may improve otitis media	Yes	188	63.9
Sinusitis in pediatric may be improved by adenotonsillectomy	Yes	121	41.2
Snoring is associated with tonsils and adenoid hypertrophy	Yes	250	85.0
Enlarged tonsils and adenoid hypertrophy may cause pediatric sleep apnea	Yes	194	66.0
Enlarged tonsils and adenoid hypertrophy can affect the tone of the voice (nasal tone)	Yes	250	85.0
Adenotonsillitis can cause bad breath (halitosis)	Yes	218	74.1
Adenotonsillitis can cause dental caries	Yes	75	25.5
Recurrent adenotonsillitis may cause weight loss	Yes	142	48.3
Chronic neglected adenotonsillitis can cause pulmonary hypertension, cor pulmonale, and heart failure	Yes	76	25.9
Gastroesophageal reflux disease can cause recurrent adenotonsillitis	Yes	79	26.9
Adenotonsillitis can present as neck swelling (cervical lymph nodes, neck abscess)	Yes	146	49.7
Tonsils can be removed while it is infected (acute follicular tonsillitis)	No	132	44.9
Immune response weakens after adenotonsillectomy	No	106	36.1
Obesity and excessive weight gain is one of the complications of adenotonsillectomy	No	125	42.5
The most common complication following adenotonsillectomy is bleeding and dehydration	Yes	141	48.0
Adenoid recurrence can occur after adenoid removal	Yes	146	49.7

**Figure 2:**
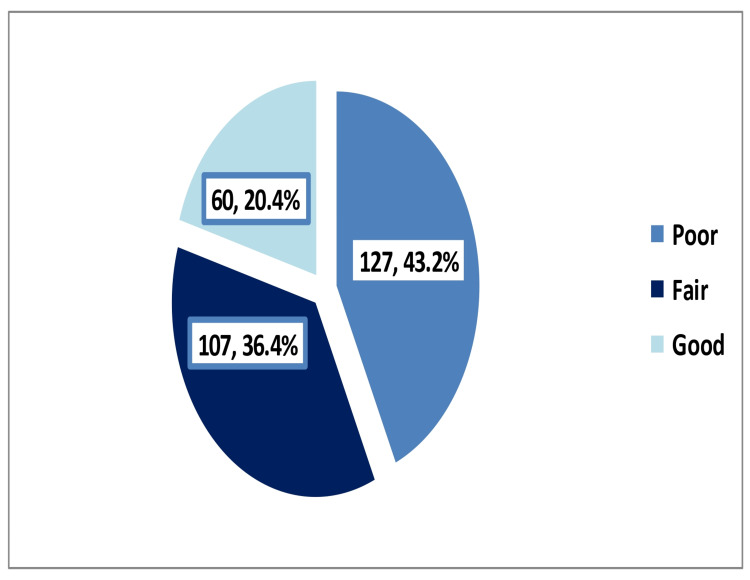
Level of parents’ knowledge regarding the complications of recurrent adenotonsillitis and its surgical treatment

Overall, 127 parents (43.2%) had a poor level of knowledge, whereas 60 (20.4%) had a good level of knowledge regarding the complications of recurrent adenotonsillitis and its surgical treatment, as shown in Figure 2.

Almost a quarter of the parents had graduate or postgraduate degrees (58; 23.1%), had a good level of knowledge regarding complications of recurrent adenotonsillitis and its surgical treatment, whereas only two (4.7%) of the secondary school or lower educated parents had the same (p < 0.001). Participants’ age, sex, and nationality were not significantly associated with their knowledge of the complications of recurrent adenotonsillitis and its surgical treatment (Table 3).

**Table 3:** Participant’s personal factors associated with their knowledge regarding the complications of recurrent adenotonsillitis and its surgical treatment *Analysis of variance (ANOVA) test

	Level of knowledge			p-value
	Poor N=127	Fair N=107	Good N=60	
Age (years)				0.531*
Mean ± SD	33.3 ± 6.4	33.3 ± 7.2	32.2 ± 7.3	
Sex No. (%)				0.216
Male (n=153)	61 (39.9)	55 (35.9)	37 (24.2)	
Female (n=141)	66 (46.8)	52 (36.9)	23 (16.3)	
Nationality No. (%)				0.249
Saudi (n=276)	117 (42.4)	100 (36.2(	59 (21.4)	
Non-Saudi (n=18)	10 (55.5)	7 (38.9)	1 (5.6)	
Educational level				<0.001
Secondary school/below (n=43)	32 (74.4)	9 (20.9)	2 (4.7)	
University/above (n=251)	95 (37.8)	98 (39.1)	58 (23.1)	

Participants with a family history of recurrent adenotonsillectomy were more knowledgeable regarding the complications of recurrent adenotonsillitis and its surgical treatment compared to those with no such history (35; 36.1% vs. 25; 12.7%, respectively; p<0.001) (Table 4).

**Table 4:** Association between family history of recurrent adenotonsillectomy among the participating parents and their knowledge regarding the complications of recurrent adenotonsillitis and its surgical treatment * Chi-square test

	Level of knowledge			p-value
	Poor	Fair	Good	
	N=127	N=107	N=60	
	N (%)	N (%)	N (%)	
No (n=197)	114 (57.9)	58 (29.4)	25 (12.7)	<0.001*
Yes (n=97)	13 (13.4)	49 (50.5)	35 (36.1)	

## Discussion

The current study revealed deficient parental knowledge regarding the complications of recurrent adenotonsillitis and its surgical treatment given that only one-fifth of the parents/guardians of children undergoing adenotonsillectomy expressed good knowledge, whereas 43.2% had demonstrated poor knowledge. The same findings have been reported by others [20]. This deficiency in knowledge was particularly prominent in questions regarding the negative impact of removal of adenoid and tonsils on the body immunity, weight gain, serious complications of long standing neglected adenotonsillitis such as pulmonary hypertension, cor pulmonale, and heart failure; and the association between gastroesophageal reflux disease and recurrent adenotonsillitis. Studies have proven that adenotonsillectomy had no impact on the body’s immunity [21]. However, one homeopathic theory has suggested that the tonsils should not be removed if possible given that they are a part of the immune system [22]. Moreover, evidence has shown that adenotonsillectomy does not cause obesity or weight gain. On the contrary, studies have proven that it is effective in reducing obstructive symptoms in obese children and improving their quality of life [23]. Chronic neglected adenotonsillitis has been associated with serious complications, such as pulmonary hypertension, cor pulmonale, and heart failure [24], with other studies showing an association between gastroesophageal reflux disease and recurrent adenotonsillitis [25].

 Most of the parents included in the present study were aware of the adverse effects of adenotonsillitis, including otitis media, snoring, nasal tone of voice, halitosis, and pediatric sleep apnea. The same has been observed in another study conducted in India among mothers of children with tonsillitis [26]. However, deficient awareness was observed concerning the link between adenotonsillitis and dental caries [27], sinusitis [28], and reduced body weight [29].

Almost half of the parents in the present study were able to recognize that the most common complication after adenotonsillectomy is bleeding and dehydration and that recurrence can occur after adenoid removal.

Although tonsillectomy has an overall positive influence on the quality of life of pediatric patients, parents of children with recurrent acute tonsillitis who are candidates for tonsillectomy must be made aware that the clinical benefits, such as decreased number of pharyngeal pain episodes, may be limited in cases with severe symptoms and non-existent in those with mild symptoms, for which non-surgical treatment should be considered [30].

As expected, more highly educated parents/guardians and those with a family history of recurrent adenotonsillectomy were more knowledgeable regarding the complications of recurrent adenotonsillitis.

To the best of our knowledge, this has been the first study within the Kingdom of Saudi Arabia to assess parents’ knowledge regarding adenotonsillitis and post-adenotonsillectomy complications in their children, subsequently showing that the beneficial effects of tonsillectomy could be ascertained and more pertinent recommendations for surgery may be formulated. However, given that this was a single-center study, our results cannot be generalized to other populations. Moreover, its cross-sectional design can only prove association and not causality between dependent and independent variables.

## Conclusions

A considerable proportion of parents/guardians of children scheduled to undergo adenotonsillectomy had poor knowledge regarding the complications of recurrent adenotonsillitis and its surgical treatment. Higher educated parents/guardians and those with family history of recurrent adenotonsillectomy were more knowledgeable than those with lesser educational attainment and no history of recurrent adenotonsillectomy. As such, health education may be an approach toward improving knowledge levels. Moreover, further larger-scale longitudinal studies are warranted to establish a more comprehensive profile of the situation in our region.

## References

[1] (2019). Physiologie des Menschen: mit Pathophysiologie. Physiologie des Menschen: mit Pathophysiologie. Berlin: Springer.

[2] Scadding GK (1990). Immunology of the tonsil: a review. J R Soc Med.

[3] Burton MJ, Glasziou PP (2009). Tonsillectomy or adeno-tonsillectomy versus non-surgical treatment for chronic/recurrent acute tonsillitis. Cochrane Database Syst Rev.

[4] Joshi S (2019). Clinical and bacteriological study of acute tonsilitis. Int J Med Sci Diag Res.

[5] Kaditis AG, Alonso Alvarez ML, Boudewyns A (2016). Obstructive sleep disordered breathing in 2- to 18-year-old children: diagnosis and management. Eur Respir J.

[6] Pabla L, Duffin J, Flood L, Blackmore K (2018). Paediatric obstructive sleep apnoea: is our operative management evidence-based?. J Laryngol Otol.

[7] Powell S, Kubba H, O'Brien C, Tremlett M (2010). Paediatric obstructive sleep apnoea. BMJ.

[8] Grippaudo C, Paolantonio EG, Antonini G, Saulle R, La Torre G, Deli R (2016). Association between oral habits, mouth breathing and malocclusion. Acta Otorhinolaryngol Ital.

[9] Shin KS, Cho SH, Kim KR, Tae K, Lee SH, Park CW, Jeong JH (2008). The role of adenoids in pediatric rhinosinusitis. Int J Pediatr Otorhinolaryngol.

[10] Stelter K (2014). Tonsillitis and sore throat in children. GMS Curr Top Otorhinolaryngol Head Neck Surg.

[11] Ravi R, Howell T (2007). Anaesthesia for paediatric ear, nose, and throat surgery. Contin Educ Anaesth Crit Care Pain.

[12] Baugh RF, Archer SM, Mitchell RB (2011). Clinical practice guideline: tonsillectomy in children. Otolaryngol Head Neck Surg.

[13] Meister KD, Messner AH (2019). Ear, nose, and throat. Pediatric Board Study Guide.

[14] Ramos SD, Mukerji S, Pine HS (2013). Tonsillectomy and adenoidectomy. Pediatr Clin North Am.

[15] Marcus CL, Moore RH, Rosen CL (2013). A randomized trial of adenotonsillectomy for childhood sleep apnea. N Engl J Med.

[16] Bangera A (2017). Anaesthesia for adenotonsillectomy: an update. Indian J Anaesth.

[17] Johnson LB, Elluru RG, Myer 3rd CM (200211282). Complications of adenotonsillectomy. Laryngoscope.

[18] Konstantinopoulou S, Gallagher P, Elden L (2015). Complications of adenotonsillectomy for obstructive sleep apnea in school-aged children. Int J Pediatr Otorhinolaryngol.

[19] Wikipedia. Medina Region (2020). Wikipedia: Medina region. https://en.wikipedia.org/wiki/Medina_Region.

[20] Roos K (1985). The diagnostic value of symptoms and signs in acute tonsillitis in children over the age of 10 and in adults. Scand J Infect Dis.

[21] Santos FP, Weber R, Fortes BC, Pignatari SS (2013). Short and long term impact of adenotonsillectomy on the immune system. Braz J Otorhinolaryngol.

[22] Soni-Jaiswal A, Anderco I, Kumar BN (2014). Patient-reported outcomes in children suffering with mild to moderate tonsillitis versus those in children with severe tonsillitis. J Laryngol Otol.

[23] Tatlıpınar A, Kınal E (2015). Links and risks associated with adenotonsillectomy and obesity. Pediatric Health Med Ther.

[24] Abdel-Aziz M (2011). Asymptomatic cardiopulmonary changes caused by adenoid hypertrophy. J Craniofac Surg.

[25] Fandiño LH, Tarazona NM, Díaz JA (2011). Reflux laryngitis: an otolaryngologist's perspective. Rev Col Gastroenterol.

[26] Sarojini K, Dinesh P (2019). Assessing the level of knowledge regarding tonsillitis and its prevention among mothers. Drug Invent Today.

[27] Al-Otaibi MK, Ahmed S, Al-Abdullah FA (2019). Bacteriological correlation between dental plaque and chronic tonsillitis. J Interdiscip Dentistry.

[28] Del Mar C (2016). Acute sinusitis and sore throat in primary care. Aust Prescr.

[29] Abazi B, Shaqiri B, Ajvazi H, Lutaj P, Radovani P (2015). Clinical impact of chronic tonsillitis on weight and height parameters. Med Arch.

[30] Pignataro L, Ibba T, Marchisio P, Torretta S (2018). Adenotonsillectomy in children with recurrent acute tonsillitis: review and implications for practice adenotonsillectomy in recurrent acute tonsillitis. Biomedical.

